# Survival estimates stratified by the Nottingham Prognostic Index for early breast cancer: a systematic review and meta-analysis of observational studies

**DOI:** 10.1186/s13643-018-0803-9

**Published:** 2018-09-15

**Authors:** Ewan Gray, Anna Donten, Katherine Payne, Peter S. Hall

**Affiliations:** 1 The University of Edinburgh, Edinburgh, UK; 2 Manchester Centre for Health Economics, The University of Manchester, Manchester, UK

**Keywords:** Breast cancer, Prognosis, Precision medicine, Meta-analysis, Nottingham Prognostic Index

## Abstract

**Background:**

Estimates of survival for women diagnosed with early staged breast cancer are available based on stratification into prognostic categories defined using the Nottingham Prognostic Index (NPI). This review aimed to identify and summarize the estimated survival statistics from separate sources in the literature and to explore the extent of between-study heterogeneity in survival estimates.

**Methods:**

Observational studies in women diagnosed with early and locally advanced breast cancer reporting overall survival by NPI category were identified using a systematic literature search. An exploratory meta-analysis was conducted to describe survival estimates and assess between-study heterogeneity.

**Results:**

Twenty-eight studies were identified. Nineteen studies with sufficient data on overall survival were included in meta-analysis. A high level of heterogeneity in survival estimates was evident with *I*^2^ values in the range of 90 to 98%.

**Conclusions:**

The substantial differences between studies in the relationship between NPI categories and survival at 5 and 10 years poses challenges for use of this prognostic score in both clinical settings and in decision-analytic model-based economic evaluations.

**Electronic supplementary material:**

The online version of this article (10.1186/s13643-018-0803-9) contains supplementary material, which is available to authorized users.

## Background

The Nottingham Prognostic Index (NPI) is a commonly used, clinically relevant and internationally validated [1, 2] system for classifying early and locally advanced breast cancer cases (TNM stages I, II, and III [3]) into three or more prognostic groups [4, 5]. Although more advanced models [6, 7] have superseded NPI in some applications, it continues to have an active role in clinical practice and research [8]. In clinical practice, NPI and related prognostic models have an important application for patients and clinicians to inform the decision of whether or not to undergo adjuvant chemotherapy following surgery [9], an early example of what is commonly called precision medicine [10].

The NPI can be used to provide prognostic information by assigning individuals into prognostic categories and then applying the survival estimates from a previous cohort study [11]. As well as providing prognostic estimates for individual patients, the NPI is useful in the context of economic evaluations. In economic evaluation of new interventions, overall survival is a key input for decision-analytic models designed to quantify the incremental costs and benefits and inform if, and how, to allocate finite healthcare budgets towards new screening and management options [12]. Decision-analytic models have a key role in the evaluation of new technologies when clinical trials are not likely to be feasible or timely in terms of producing robust evidence of the impact of introducing new programmes or changing existing programmes [13].

The NPI categories are a linear combination of three prognostic factors: tumour size (maximum diameter in millimetres), histological grade (1- to 3-point scale) and lymph node staging (1- to 3-point scale). Standardized published cut-offs (see Additional file 1: Appendix 1) are used to form prognostic categories within a population (e.g. good, moderate and poor prognostic groups).

This study aimed to identify and describe all published observational studies reporting NPI-category-specific overall survival following a diagnosis of early and/or locally advanced breast cancer. A meta-analysis was used to explore the between-study heterogeneity in survival estimates.

## Methods

A systematic review and meta-analysis, following published recommendations [14, 15], were used to identify all published cohort studies investigating the survival of early and locally advanced breast cancer patients stratified into prognostic groups using the NPI and to synthesize the results. This systematic review was not registered with PROSPERO.

### Data sources and searches

Two databases (Embase: date of inception 1974 to 9 November 2016; MEDLINE: date of inception 1946 to 9 November 2016) were searched using bespoke electronic search strategies (see Additional file 1: Appendix 2) informed by search criteria designed by Nelson et al. [16]. Hand searching of reference lists of included studies was also undertaken.

### Study selection

Retrieved titles and abstracts were screened independently by two reviewers (EG, AD) in accordance with the inclusion and exclusion criteria. The review includes all cohort studies^1^ of women with early or locally advanced breast cancer, reporting all-cause mortality (overall survival) stratified by NPI group. Only journal articles or reports published in English were included. Studies were excluded if they were limited to only a specified sub-group of the full population (e.g. HER-2-positive patients only) or if they included patients with recurrent cancer^2^ or *ductal carcinoma* in situ.^3^ To be included in the meta-analysis, a study must have reported survival estimates as tables of survivors/events per year or graphically as survival curves.

### Quality assessment and data extraction

The Critical Appraisal Skills Programme (CASP) Cohort Study Checklist [17] was used to appraise the reporting quality of the selected studies (see Additional file 1: Appendix 3). The following data were extracted and tabulated by two reviewers: study setting, sample description, methods of analysis and survival estimates (‘overall survival’). Overall survival data were presented in identified studies as tables or graphs. Survival data from the studies using graphs were extracted using visual assessment. Guyot et al. suggest the use of algorithms for extracting data from digitized curves and conversion to time-to-event data using inverted Kaplan-Meier equations. Guyot et al.’s method was not used in this study because it requires more detailed reporting of numbers at risk (at multiple time points) than what was generally available in the included studies. Annual survival estimates and initial sample sizes were used recorded (see Additional file 1: Appendix 4 for further details of data extraction). Reconstruction of individual level time-to-event data from published survival curves has been suggested to improve the precision of meta-analysis of survival estimates. The advantages are that is potentially possible to include information regarding the censoring of observations [18] and combine survival data reported over differing lengths of follow-up.

### Data analysis

All statistical analyses were conducted using Stata version 14. Forest plots were used to present individual study survival estimates for both 5 years and 10 years of follow-up. These are ordered from most distant to most recent year of mid-point of data collection. Meta-analysis, using both fixed and random effects [19] models, produced pooled estimates of both 5-year and 10-year survival, for each NPI category separately, and allowed calculation of between-study heterogeneity statistics.

Heterogeneity between-study included datasets was assessed primarily using the *I*^2^ statistic because the interpretation is appropriate and useful from an exploratory and hypothesis generating perspective [20]. This statistic is an approximate measure of what part of the variance between the estimated effects in the meta-analysis is caused by study heterogeneity rather than sampling error. An *I*^2^ score over 75% is typically taken to indicate high heterogeneity. The statistic describes the observed heterogeneity and should not be used to make inference regarding the range of true effects [21]. The *Q* statistic provides a statistical test of the null hypothesis that studies are homogenous (all estimating the same true effect). However, it should be noted that statistical test may be an underpowered test for heterogeneity when the number of studies is small [22].

The tau statistic was also considered, which provides an estimate of the between-study standard deviation in the true effects within a random effects meta-analysis framework [23]. The choice between using the fixed effects or the random effects meta-analysis depends on the assumed perspective regarding the underlying true effect(s) and the desired interpretation of the precision weighted average survival estimate [24]. Assumptions about the underlying true effects for these data are discussed in the terminology of Spiegelhalter et al. [25]: ‘identical parameters’, ‘independent parameters’ or ‘exchangeable parameters’. The ‘identical parameters’ assumption is probably unrealistic in this setting given the diversity in study circumstances. Assuming ‘independent parameters’ may be reasonable if the variation between studies is caused by a variety of unique differences in specific settings, and the ‘exchangeable parameters’ assumption may also be reasonable if the variation between studies can be described by a single mixing distribution. An independent parameters assumption would motivate a fixed effects meta-analysis, while exchangeable parameters would motivate a random effects meta-analysis. In this exploratory study, we are ambivalent about which of these assumptions is the most appropriate, believing both may be reasonable, and therefore present both fixed and random effects estimates.

## Results

**Fig. 1:**
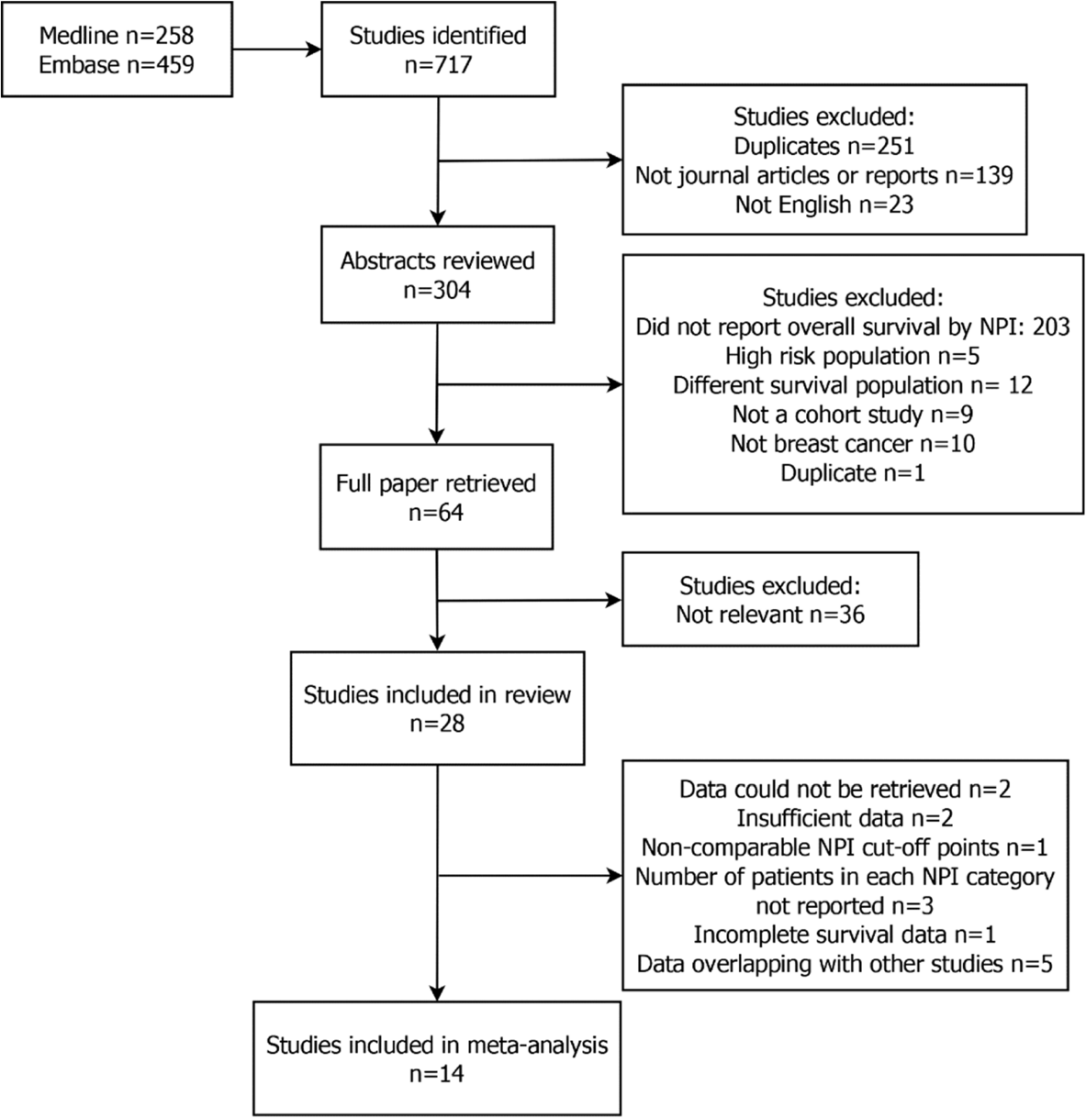
Study selection process

**Fig. 2:**
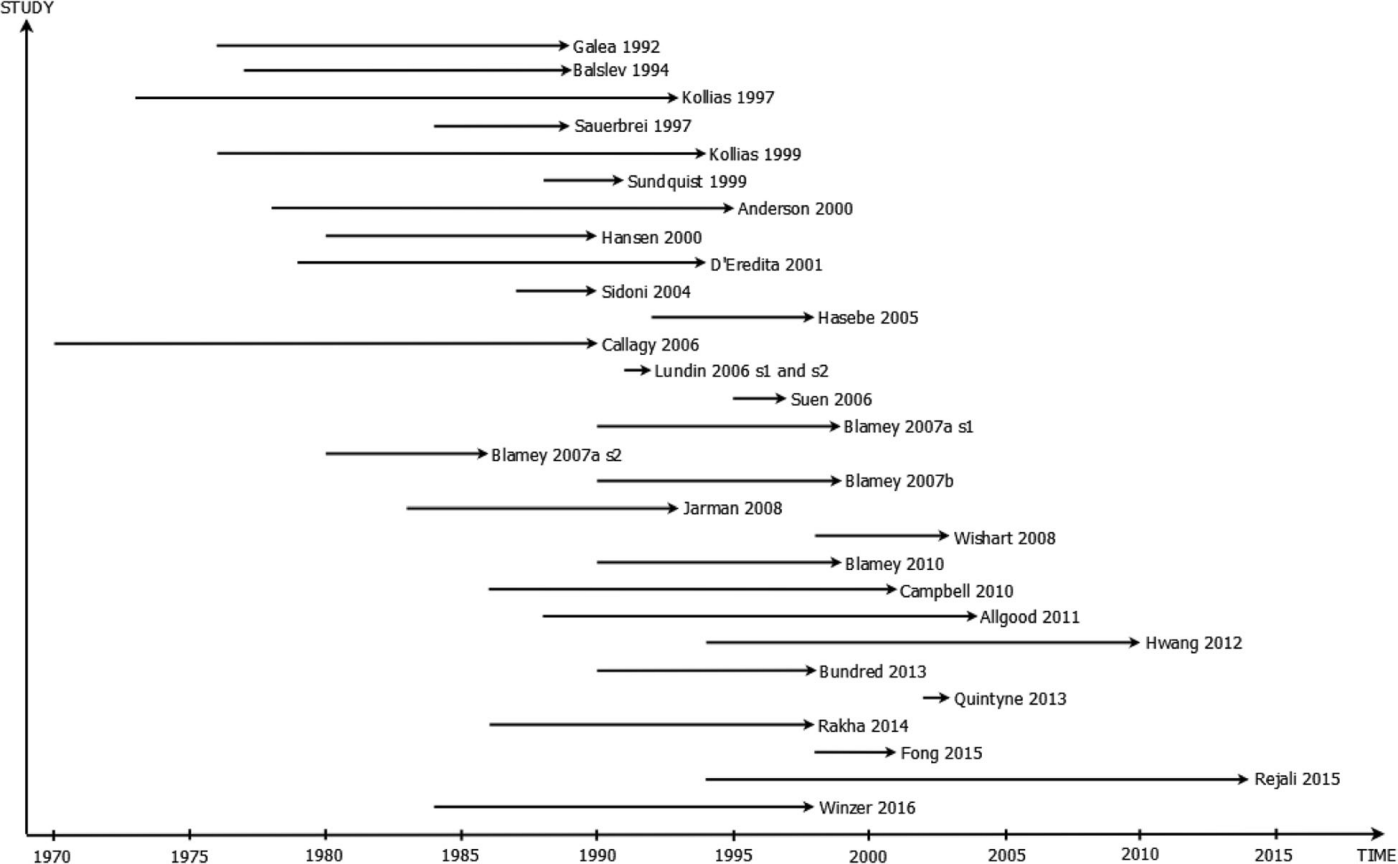
Summary of time points for data collection (recruitment) for each dataset. Studies presented in order of publication date

A total of 28 studies were suitable for inclusion in the review (see Fig. 1) and 19 of these studies were included in the meta-analysis (see Table 1). Of the 28 studies, two studies [2, 26] provided the results of survival analysis for two data series; therefore, a total of 30 datasets were included in the review and 20 in the meta-analysis. Three studies [27–29] provided only data up to 5-year survival and were excluded from the meta-analysis of 10-year survival. Nine studies (containing ten datasets) were excluded from any kind of meta-analysis due to the following reasons. The quality of the graphical presentation in two studies [30, 31] did not allow for extraction of the survival data. Three further studies [2, 32, 33] did not report numbers of patients in each NPI category. One [34] used different cut-off points for NPI that meant the categories were not comparable with the majority of the studies. Two studies [35, 36] reported insufficient data. One study [37] excluded patients who died in the first months after the surgery, and therefore, the provided survival estimates were incomplete. Eight data series from seven studies [1, 5, 26, 38–41] reported results from the original Nottingham case series and/or West Midlands Cancer registry (to which Nottingham contributes). These studies report partially overlapping cohorts of patients (see Fig. 2). To minimize ‘double-counting’, four studies were excluded from the meta-analysis which meant that the remaining included studies [1, 5, 41] contained minimal overlap in the reported cohorts. Among the remaining studies, Blamey et al. [1] does overlap with Allgood et al. [41]; however, the proportion of data that was shared was a relatively small proportion of the data because the first study reports a pooled European cohort of which approximately 15% of the data is from Nottingham, while the second study reports data from the West Midlands Cancer Registry to which Nottingham contributes only a minority of cases.

**Table 1:** Summary of studies included in the review and meta-analysis

Study details		
Characteristic	Systematic review	Meta-analysis
	Number of data series (*n* = 30)	Number of data series (*n* = 14)
Location		
UK only	14	6
Europe	10	5
USA	1	0
Other	5	3
Size of sample		
< 100 women	2	1
100 to 1000 women	10	6
1000 to 10,000 women	15	5
> 10,000 women	3	2
NPI categories		
Three	18	8
Five	2	2
Other	10	4
Length of analysis		
5 years	4	2
Between 5 and 10 years	3	1
10 years or more	23	11
Type of analysis		
Parametric	4	0
Cox proportional hazard model	20	9
Other (Kaplan-Meier estimator)	6	5

Note: One study [1] reported data from ten European countries including the UK; therefore, it is classified as ‘European’ study, not ‘UK only’

### Description of studies

The earliest data reported in the published studies came from 1970 [42] and most recent data from 2014 [43] (see Fig. 2). Fourteen of the identified data series (47%) reported UK data. Ten studies reported data from other European countries, and one study [1] reported data from ten European countries, including the UK. One study [2] compared two datasets: from Finland and the USA. Single studies reported data from each of Canada [42], Japan [35], South Korea [32], Hong Kong [27] and Iran [43]. Study reporting quality was generally assessed as satisfactory, and risk of bias in relation to the study survival estimates was considered to be low for all included studies (see Additional file 1: Appendix 3).

Start of each line represents first time point of data collection, and arrow head represents last time point of data collection for each study dataset.

### Systematic review of evidence

There was diversity between the included studies in how women with breast cancer were classified by NPI category. The majority of the studies (*n* = 18; 60%) used three NPI-categories. Two studies [29, 44] (7%) grouped the sample into five NPI categories. The remaining ten (33%) studies used other numbers of NPI groups ranging from four to ten (see Table 1). Given the majority of studies used three NPI categories, the data from all studies were aggregated into three groups collapsing NPI categories where necessary. Three studies [30, 42, 45] did not provide cut-off points for NPI categories, and it was, therefore, assumed these studies used the standard cut-off values: NPI less than 3.4 for ‘good prognostic group’, between 3.4 and 5.4 for ‘moderate prognostic group’ and greater than 5.4 for ‘poor prognostic group’. Two studies [28, 41] provided different cut-off points. It was assumed that observations with NPI less than 3.41 belong to the good prognostic group and observations with NPI less than 5.41 in the Jarman et al. [28] study and NPI less than 5.28 in Allgood et al. [41] study belong to the moderate prognostic group.

**Fig. 3:**
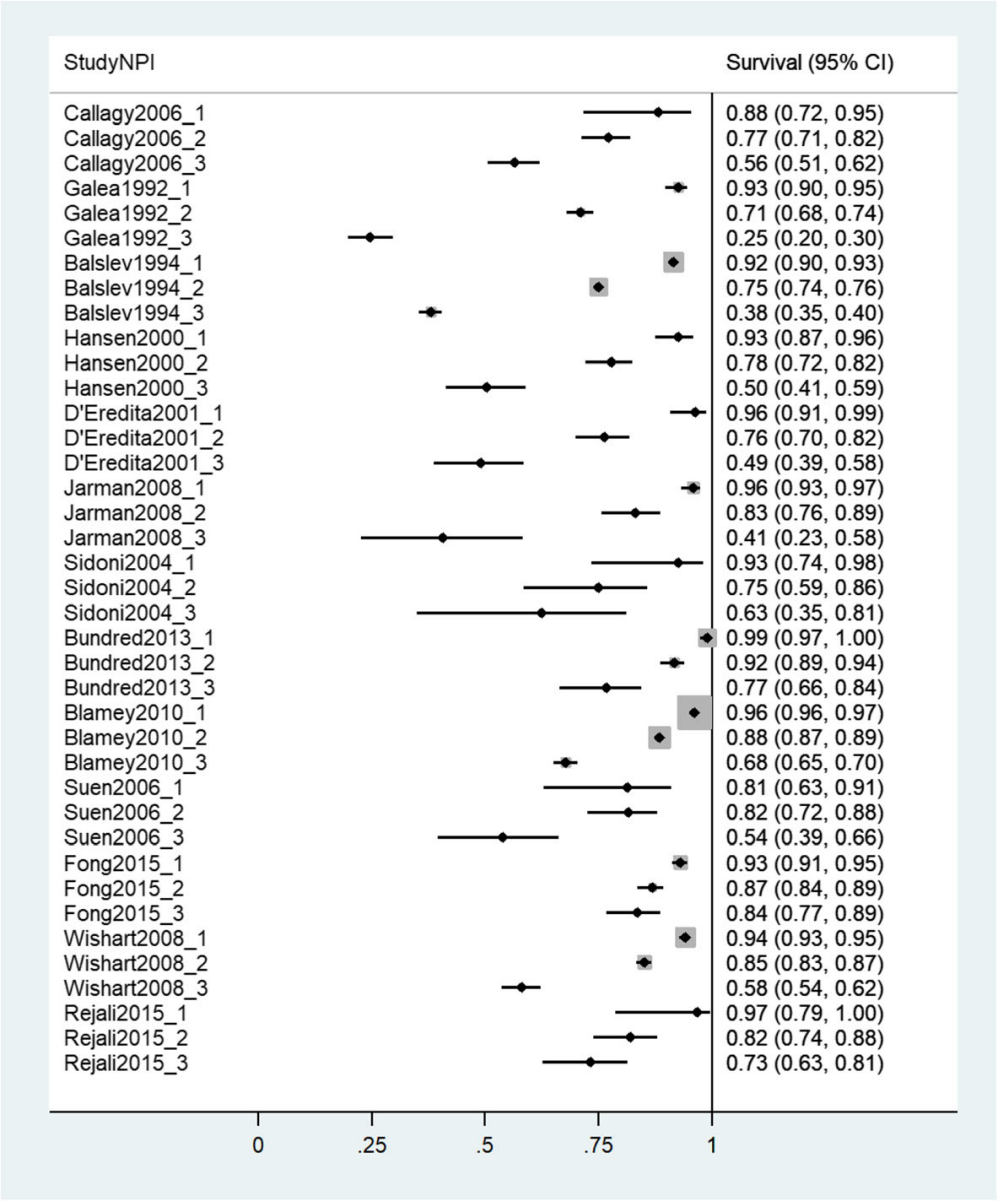
Forest plot of 5-year survival estimates from individual studies. Studies presented in order of mid-point of data collection earliest to most recent. *X*-axis shows proportions of sample surviving at the specified time point

**Fig. 4:**
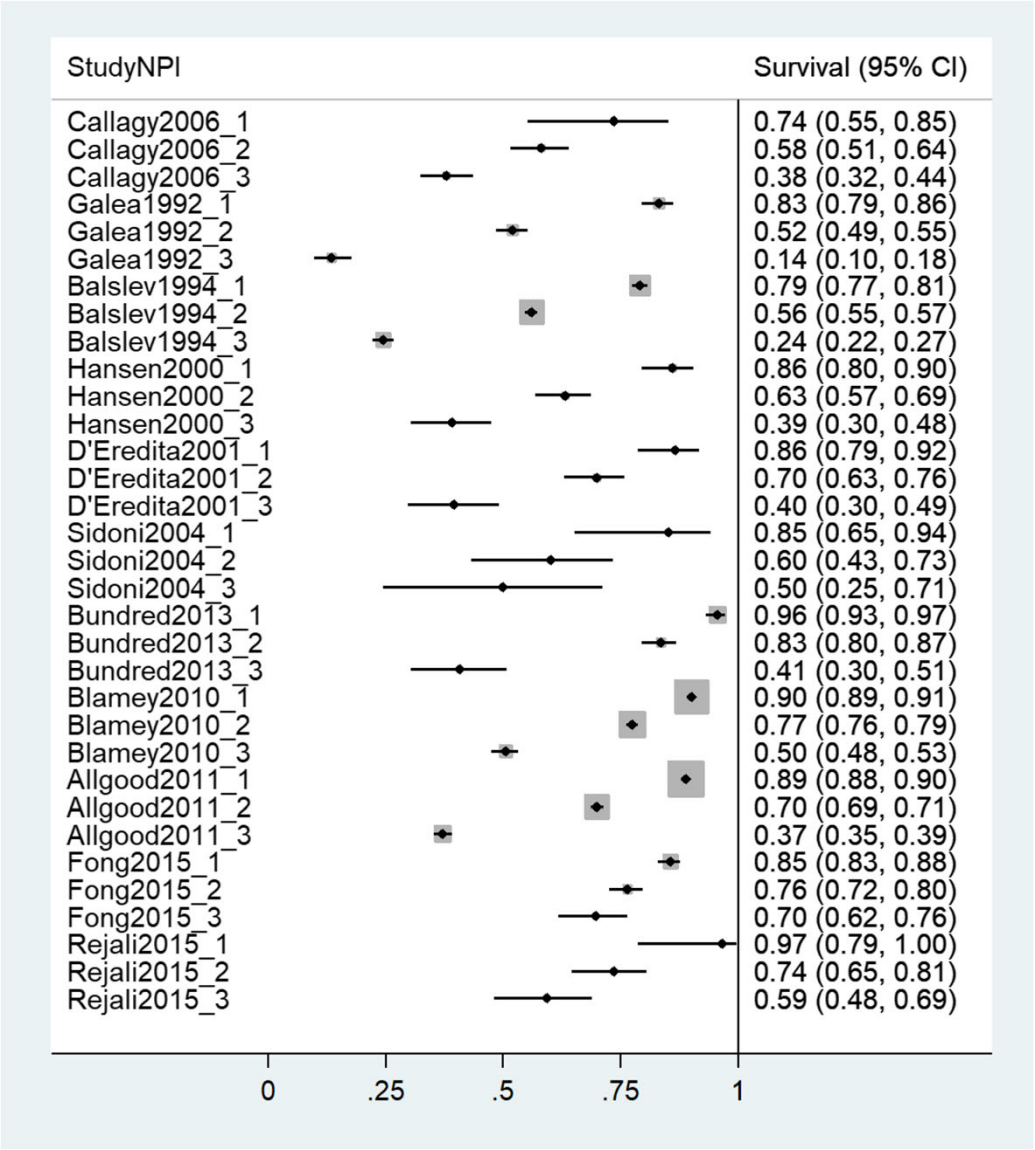
Forest plot of 10-year survival estimates from individual studies. Studies presented in order of mid-point of data collection earliest to most recent. *X*-axis shows proportions of sample surviving at the specified time point

Figures 3 and 4 report the percentage of women surviving with breast cancer at two time points, 5 years and 10 years respectively, for the available datasets from which at least one of these statistics were available (Additional file 1: Appendix 5 for numbers in each study). One study [27] only reported survival data up to 9 years. Three studies [29, 40, 41] only provided survival data for the final year of analyses in each respective study.

The forest plots provide a clear visualisation of the trends between NPI categories within studies and, as these are ordered by the mid-point of data collection, differences in overall survival across time periods. The observed pattern of overall survival by NPI category was consistent across all datasets. Overall survival was seen to be noticeably worse in NPI category 3 compared with NPI categories 1 and 2. Visual inspection suggests substantial heterogeneity was observed in overall survival between the available datasets. To illustrate, 5-year survival for NPI category 1 was as low as 81.25% [27] but also as high as 99.26% [8]. Similarly, for NPI category 3, the lowest 5-year overall survival was 24.56% [5] and the highest was 83.55% [46].

### Meta-analysis

The results of the meta-analysis are presented in Table 2 for both 5-year survival and 10-year survival statistics. There was evidence of considerable heterogeneity in the estimated survival statistics between the included datasets. The *I*^2^ statistic indicated high heterogeneity for each of the NPI category with approximately 90 to 98% of the between-study variation estimated as being due to heterogeneity rather than sampling variation, suggesting pooling of survival estimates was inappropriate in all NPI categories. Heterogeneity was also considered present based on *Q* statistics which were statistically significant in all instances. Tau statistics provide further evidence for substantial heterogeneity with the reported standard deviations of effect size being 2, 7 and 18 percentage points (categories 1 to 3 respectively) for 5-year survival and 5, 11 and 14 percentage points for 10-year survival.

**Table 2:** Results from the meta-analysis, pooled survival estimates and heterogeneity statistics

Statistic	NPI 1	NPI 2	NPI 3
5-year follow-up			
Fixed effects I-V estimate (95% CI)	0.953 (0.949, 0.957)	0.831 (0.825, 0.837)	0.535 (0.521, 0.549)
Random effects D-L estimate (95% CI)	0.943 (0.927, 0.96)	0.811 (0.769, 0.853)	0.565 (0.462, 0.668)
*I*^2^ (%)	89.6	97.2	97.7
*Q*	115.93 (*P* < 0.001)	422.5 (*P* < 0.001)	532.32 (*P* < 0.001)
Tau (% points)	2.45	7.28	18.25
10-year follow-up			
Fixed effects I-V estimate (95% CI)	0.883 (0.877, 0.888)	0.682 (0.675, 0.689)	0.353 (0.341, 0.364)
Random effects D-L estimate (95% CI)	0.869 (0.837, 0.901)	0.674 (0.608, 0.74)	0.414 (0.329, 0.499)
*I*^2^ (%)	95.3	98.6	97.7
*Q*	213.56 (*P* < 0.001)	727.14 (*P* < 0.001)	427.44 (*P* < 0.001)
Tau (% points)	4.58	10.77	13.71

*I-V* inverse variance, *D-L* DerSimonian and Laird method

## Discussion

This review identified a substantial number of observational studies that aimed to generate estimates of the overall survival of women diagnosed with early and/or locally advanced breast cancer stratified by prognostic category using NPI.

The strengths of this study include using a comprehensive, systematic review of the literature underpinned by robust review methods (see Additional file 2: PRISMA checklist). A broad electronic search strategy, supplemented with hand searching of reference lists of identified studies, to collate all the relevant studies and datasets. The search strategy and inclusion criteria achieved good sensitivity in identifying relevant studies. The identified studies included patient cohorts from a broad range of time periods for data collection, countries and age groups. Critical appraisal following a standard protocol found study reporting quality to be generally satisfactory. A number of relevant identified studies had to be excluded from the meta-analysis of overall survival as the relevant data could not be extracted from the published manuscript and authors were not able to provide the data required. The studies excluded at this stage did not appear to suggest different outcomes for survival compared to the included studies based on the more limited information available. We focussed on cohort studies and did not include randomized controlled trials (RCT). RCT studies were considered unlikely to report NPI-stratified survival estimates and are likely to have applied more extensive exclusion criteria than cohort studies. No formal quantitative assessment of publication bias was made. It is not clear in this context the mechanism or result of publication bias that should be expected because each study was testing different hypothesis/hypotheses and in which the data extracted for this study were presented descriptively.

A particular concern in evidence synthesis is whether it was valid to pool data across multiple studies [20].This exploratory meta-analysis investigated study heterogeneity and demonstrated that this was too severe for simple pooling of survival estimates to be appropriate for the purposes of estimating survival in the current population. It is of interest to understand the factors driving the heterogeneity in survival estimates; however, these data are insufficient to allow a robust meta-regression or multilevel analysis to test hypotheses about such determinants. There are only a small number of studies and many possible explanatory variables, including some for which only weak surrogate markers would be available from study level data. Investigation of the sources of between-study heterogeneity could be best achieved using an individual patient data meta-analysis to explore factors such as the year of diagnosis of patients, country, screen detection rate and age.

This study had a number of limitations. To provide quantitative estimates of overall survival by NPI category, given the relative age of some of the identified studies, it was necessary to collate some data by visual inspection of published survival curves. This process will have introduced some measurement error. Due to lack of detailed reporting of losses to follow-up it was also necessary to assume that all studies had no loss to follow-up, which could result in different degrees of selection bias in the results if there were differential losses to follow-up across studies. Based on the published study protocols and the routine and often statutory collection of mortality data, we believe that losses to follow-up are likely to be minimal. Due to limitations of available resources, non-English language studies and those with insufficient reporting of data were excluded. Furthermore, it was considered infeasible based on available resources and likely data sharing restrictions, to attempt to collate individual patient data to address some of these limitations. The resulting exclusions may introduce some selection bias, but it is not possible to know the impact of such a bias.

There were some observed differences in included studies in the methods used to assess lymph node status which is a key input to define NPI category. Originally, node involvement for selected NPI category was assessed using biopsy results of a lower axillary node, an apical axillary node and a node from the internal mammary chain. Node status was specified as one if the tumour was absent from all three nodes, two if tumour cells were found in lower axillary node only, and three if tumour cells were in the apical and/or internal mammary node [4]. To increase usability of the NPI, Galea et al. [5] suggested staging based on the number of involved nodes from the single location as a viable alternative, which was subsequently used in later studies. However, we believe this change in assessment of node status was sufficiently similar to not introduce bias in the analysis.

## Conclusions

Precision medicine promises to deliver improved patient outcomes through better understanding of patient and disease heterogeneity, and ultimately better targeted screening and therapeutic strategies. Moving the concept of precision medicine into clinical practice requires mechanisms to identify and stratify eligible patient populations. Prognostic models are an important component in such a mechanism because they allow an individualized quantitative estimate of potential treatment benefit.

This systematic review and meta-analysis of NPI stratified estimates of survival revealed a key challenge in the design and evaluation of precision medicine interventions; that substantial differences may exist between studies estimating the relationship between a marker or score and the outcome of interest. Heterogeneity of study estimates should be investigated carefully for all such interventions. This finding suggests a potential additional burden on shared decision-making between clinicians and patients. Achieving a better informed decision is only possible if all of the available evidence is correctly synthesized and clearly presented.

The results of this systematic review and meta-analysis have implications for patients, clinicians, decision analysts and policy analysts requiring a prediction of long-term overall survival, in women diagnosed with early and/or locally advanced breast cancer. The observed heterogeneity in overall survival estimates indicates that it is important to make use of survival data from an appropriate setting to provide reliable prognostic information for use in clinical practice, and also in decision-analytic model-based economic evaluations of new treatments, screening programmes or prevention strategies to inform health care resource allocation decisions.

## Additional files

Additional file 1: **Appendix 1.** NPI category cut-offs. **Appendix 2.** Electronic search strategies. **Appendix 3.** Critical appraisal of accepted studies. **Appendix 4.** Data extraction procedures. **Appendix 5.** Reported five and ten-year survival per study dataset. (DOCX 71 kb)

Additional file 2: PRISMA checklist. (DOCX 21 kb)

## Abbreviations

CASP: Critical Skills Appraisal Programme
HER-2: Human epidermal growth factor receptor 2
NPI: Nottingham Prognostic Index
PROSPERO: International prospective register of systematic reviews
RCT: Randomized controlled trial
TNM: Tumour, nodes, metastases

## Footnotes

1 A study that takes a group (cohort) of patients and follows their progress over time in order to measure outcomes such as disease or mortality rates and make comparisons according to the treatments or interventions that patients received [47].

2 Cancer reappearing after a remission [48].

3 A proliferation of malignant-appearing cells of the ducts and terminal lobular units of the breast that have not breached the ductal basement membrane [49].
