# Supplementary material for: Survival estimates stratified by the Nottingham Prognostic Index for early breast cancer: a systematic review and meta-analysis of observational studies
Source: Syst Rev. 2018 Sep 15;7:142. doi: 10.1186/s13643-018-0803-9 (PMC6138917; doi:10.1186/s13643-018-0803-9)
Supplement: Supplementary file 1 — Appendix 1. NPI category cut-offs. Appendix 2. Electronic search strategies. Appendix 3. Critical appraisal of accepted studies. Appendix 4. Data extraction procedures. Appendix 5. Reported five and ten-year survival per study dataset. (DOCX 71 kb) [file 13643_2018_803_MOESM1_ESM.docx]

**Additional file 1**

Appendix 1: NPI category cut-offs

Appendix 2: Electronic search strategies

Appendix 3: Critical appraisal of accepted studies

Appendix 4: Data extraction procedures

Appendix 5: Reported five and ten-year survival per study dataset

**Appendix 1:** **NPI category cut-offs**

Three categories classification:

1 - Good Prognostic Group: $NPI\leq3.4$

2 – Moderate Prognostic Group: $3.4<NPI\leq5.4$

3 – Poor Prognostic Group: $NPI>5.4$

Five categories classification:

1 – Very Good Prognostic Group: $NPI\leq2.4$

2 - Good Prognostic Group: $NPI\leq3.4$

3 – Moderate Prognostic Group 1: $3.4<NPI\leq4.4$

4 – Moderate Prognostic Group 2: $4.4<NPI\leq5.4$

5 – Poor Prognostic Group: $NPI>5.4$

**Appendix 2:** Electronic search strategies

Medline

1. ((Breast* or mammary) adj3 (Neoplas$ or tumor$ or cancer$ or carcinoma$)).mp.
2. exp Breast Neoplasms/
3. 1 or 2
4. exp Life Expectancy/
5. exp Prognosis/
6. exp Mortality, Premature/ or exp Mortality/
7. exp Survival Analysis/ or exp Survival/ or exp Survival Rate/
8. 4 or 5 or 6 or 7
9. (NPI or "Nottingham Prognostic Index").ti,ab.
10. 3 and 8 and 9

Embase

1. ((Breast* or mammary) adj3 (Neoplas$ or tumor$ or cancer$ or carcinoma$)).mp.
2. exp breast cancer/
3. exp survival time/ or exp cancer survival/ or exp overall survival/ or exp survival/ or exp survival rate/ or exp cancer specific survival/ or exp cause specific survival/ or exp long term survival/ or exp survival prediction/ or exp short term survival/ or exp disease specific survival/
4. exp mortality/ or exp cancer mortality/ or exp premature mortality/
5. exp life expectancy/
6. prognosis/ or cancer prognosis/
7. exp prognosis/ or exp cancer prognosis/
8. (NPI or "Nottingham Prognostic Index").ti,ab.
9. 1 or 2
10. 3 or 4 or 5 or 6 or 7
11. 8 and 9 and 10

**Appendix 3:** Critical appraisal of accepted studies. Criteria from The Critical Appraisal Skills Programme (CASP) Cohort Study Checklist^17^.

|  | **Allgood 2011** | **Anderson 2000** | **Balslev 1994** | **Blamey 2007** |
| --- | --- | --- | --- | --- |
| Did the study address a clearly focused issue? | Yes | Yes | Yes | Yes |
| In terms of the population studied? | Yes | Yes | Yes | Yes |
| In terms of the risk factors studied? | Yes | Yes | Yes | Yes |
| In terms of the outcomes considered? | Yes | Yes | Yes | Yes |
| Is it clear whether the study tried to detect a beneficial or harmful effect? | Yes | Yes | Yes | Yes |
| Was the cohort recruited in an acceptable way? | Yes | Yes | No | Yes |
| Was the cohort representative of a defined population? | Yes | Yes | Yes | Yes |
| Was there something special about the cohort? | No | No | No | No |
| Was everybody included who should have been included? | Can't tell | Yes | Yes | Yes |
| Was the exposure accurately measured to minimise bias? | Yes | No | Yes | Yes |
| Did they use objective measurements? (No if subjective.) | Yes | Yes | Yes | Yes |
| Do the measurements truly reflect what you want them to (have they been validated)? | Yes | Yes | Yes | Yes |
| Were all the subjects classified into exposure groups using the same procedure? | Yes | Yes | Yes | Yes |
| Was the outcome accurately measured to minimise bias? | Yes | Yes | Can't tell | Yes |
| Did they use objective measurements? (No if subjective.) | Yes | Yes | Yes | Yes |
| Do the measurements truly reflect what you want them to (have they been validated)? | Yes | Yes | Yes | Yes |
| Has a reliable system been established for detecting all the cases (for measuring disease occurrence)? | Can't tell | Can't tell | Can't tell | Yes |
| Were the measurement methods similar in the different groups? | Yes | Yes | Yes | Yes |
| Were the subjects and/or the outcome assessor blinded to exposure (does this matter)? | No | No | No | No |
| Was the follow up of subjects complete enough? | Can't tell | Yes | Can't tell | Yes |
| Was the follow up of subjects long enough? | Can't tell | Yes | Yes | Yes |
| Did the good or bad effects have had long enough to reveal themselves? | Can't tell | Yes | Yes | Yes |
| Do you think the persons that are lost to follow-up had different outcomes than those available for assessment? | No | No | No | Can't tell |
| In an open or dynamic cohort, was there anything special about the outcome of the people leaving, or the exposure of the people entering the cohort? | No | No | No | No |
| Do you believe the results? | Yes | Can't tell | Yes | Yes |
|  | **Blamey 2007b** | **Blamey 2010** | **Bundred 2013** | **Callagy 2006** |
| Did the study address a clearly focused issue? | Yes | Yes | Yes | Yes |
| In terms of the population studied? | Yes | Yes | Yes | Yes |
| In terms of the risk factors studied? | Yes | Yes | Yes | Yes |
| In terms of the outcomes considered? | Yes | Yes | Yes | Yes |
| Is it clear whether the study tried to detect a beneficial or harmful effect? | Yes | Yes | Yes | Yes |
| Was the cohort recruited in an acceptable way? | Yes | Yes | Yes | Can't tell |
| Was the cohort representative of a defined population? | Yes | Yes | Yes | Can't tell |
| Was there something special about the cohort? | No | No | No | Yes |
| Was everybody included who should have been included? | Yes | Yes | Yes | Can't tell |
| Was the exposure accurately measured to minimise bias? | Yes | Yes | Yes | Yes |
| Did they use objective measurements? (No if subjective.) | Yes | Yes | Yes | Yes |
| Do the measurements truly reflect what you want them to (have they been validated)? | Yes | Yes | Yes | Yes |
| Were all the subjects classified into exposure groups using the same procedure? | Yes | Yes | Yes | Yes |
| Was the outcome accurately measured to minimise bias? | Yes | Yes | Yes | Can't tell |
| Did they use objective measurements? (No if subjective.) | Yes | Yes | Yes | Yes |
| Do the measurements truly reflect what you want them to (have they been validated)? | Yes | Yes | Yes | Yes |
| Has a reliable system been established for detecting all the cases (for measuring disease occurrence)? | Yes | Yes | Yes | Can't tell |
| Were the measurement methods similar in the different groups? | Yes | Yes | Yes | Yes |
| Were the subjects and/or the outcome assessor blinded to exposure (does this matter)? | No | No | No | No |
| Was the follow up of subjects complete enough? | Yes | Yes | Yes | Yes |
| Was the follow up of subjects long enough? | Yes | Yes | Yes | Yes |
| Did the good or bad effects have had long enough to reveal themselves? | Yes | Yes | Yes | Yes |
| Do you think the persons that are lost to follow-up had different outcomes than those available for assessment? | No | No | No | No |
| In an open or dynamic cohort, was there anything special about the outcome of the people leaving, or the exposure of the people entering the cohort? | No | No | No | No |
| Do you believe the results? | Yes | Yes | Yes | Can't tell |

|  | **Campbell 2010** | **D'Eredita 2001** | **Fong 2015** | **Galea 1992** |
| --- | --- | --- | --- | --- |
| Did the study address a clearly focused issue? | Yes | Yes | Yes | Yes |
| In terms of the population studied? | Yes | Yes | Yes | Yes |
| In terms of the risk factors studied? | Yes | Yes | Yes | Yes |
| In terms of the outcomes considered? | Yes | Yes | Yes | Yes |
| Is it clear whether the study tried to detect a beneficial or harmful effect? | Yes | Yes | Yes | Yes |
| Was the cohort recruited in an acceptable way? | Yes | Yes | Yes | Yes |
| Was the cohort representative of a defined population? | Yes | Yes | Yes | Yes |
| Was there something special about the cohort? | No | No | No | No |
| Was everybody included who should have been included? | Yes | Yes | No | Yes |
| Was the exposure accurately measured to minimise bias? | Yes | Yes | Yes | Yes |
| Did they use objective measurements? (No if subjective.) | Yes | Yes | Yes | Yes |
| Do the measurements truly reflect what you want them to (have they been validated)? | Yes | Yes | Yes | Yes |
| Were all the subjects classified into exposure groups using the same procedure? | Yes | Yes | Yes | Yes |
| Was the outcome accurately measured to minimise bias? | Can't tell | Can't tell | Yes | Can't tell |
| Did they use objective measurements? (No if subjective.) | Yes | Yes | Yes | Yes |
| Do the measurements truly reflect what you want them to (have they been validated)? | Yes | No^[[1]](#footnote-1)^ | Yes | Can't tell |
| Has a reliable system been established for detecting all the cases (for measuring disease occurrence)? | Can't tell | Can't tell | Yes | Can't tell |
| Were the measurement methods similar in the different groups? | Yes | Yes | Yes | Yes |
| Were the subjects and/or the outcome assessor blinded to exposure (does this matter)? | No | No | No | No |
| Was the follow up of subjects complete enough? | Yes^[[2]](#footnote-2)^ | Yes | Yes | Can't tell |
| Was the follow up of subjects long enough? | Yes | Yes | Yes | Can't tell^[[3]](#footnote-3)^ |
| Did the good or bad effects have had long enough to reveal themselves? | Yes | Yes | Yes | Can't tell |
| Do you think the persons that are lost to follow-up had different outcomes than those available for assessment? | No | No | Can't tell | Can't tell |
| In an open or dynamic cohort, was there anything special about the outcome of the people leaving, or the exposure of the people entering the cohort? | No | No | No | No |
| Do you believe the results? | Yes | Yes | Yes | Yes |

|  | **Hansen 2000** | **Hasebe 2005** | **Hwang 2012** | **Jarman 2008** |
| --- | --- | --- | --- | --- |
| Did the study address a clearly focused issue? | Yes | Yes | Yes | Yes |
| In terms of the population studied? | Yes | Yes | Yes | Yes |
| In terms of the risk factors studied? | Yes | Yes | Yes | Yes |
| In terms of the outcomes considered? | Yes | Yes | Yes | Yes |
| Is it clear whether the study tried to detect a beneficial or harmful effect? | Yes | Yes | Yes | Yes |
| Was the cohort recruited in an acceptable way? | Yes | Yes | Yes | Yes |
| Was the cohort representative of a defined population? | Yes | Yes | Yes | Can't tell |
| Was there something special about the cohort? | No | No | No | No |
| Was everybody included who should have been included? | Yes | Can't tell | Yes | Yes |
| Was the exposure accurately measured to minimise bias? | Yes | Yes | Can't tell | Yes |
| Did they use objective measurements? (No if subjective.) | Yes | Yes | Yes | Yes |
| Do the measurements truly reflect what you want them to (have they been validated)? | Yes | Yes | No | Yes |
| Were all the subjects classified into exposure groups using the same procedure? | Yes | Yes | Yes | Yes |
| Was the outcome accurately measured to minimise bias? | No | Yes | Can't tell | Can't tell |
| Did they use objective measurements? (No if subjective.) | Yes | Yes | Yes | Yes |
| Do the measurements truly reflect what you want them to (have they been validated)? | Yes | Yes | Yes | Yes |
| Has a reliable system been established for detecting all the cases (for measuring disease occurrence)? | Can't tell | Yes | Can't tell | Can't tell |
| Were the measurement methods similar in the different groups? | Yes | Yes | Yes | Yes |
| Were the subjects and/or the outcome assessor blinded to exposure (does this matter)? | No | No | No | No |
| Was the follow up of subjects complete enough? | Yes | Can't tell | Can't tell | Yes |
| Was the follow up of subjects long enough? | Yes | Yes | Can't tell | Yes |
| Did the good or bad effects have had long enough to reveal themselves? | Yes | Yes | Can't tell | Yes |
| Do you think the persons that are lost to follow-up had different outcomes than those available for assessment? | Yes | Can't tell | Can't tell | No |
| In an open or dynamic cohort, was there anything special about the outcome of the people leaving, or the exposure of the people entering the cohort? | No | No | No | No |
| Do you believe the results? | Yes | Yes | Can't tell | Yes |

|  | **Kollias 1997** | **Kollias 1999** | **Lundin 2006** | **Quintyne 2013** |
| --- | --- | --- | --- | --- |
| Did the study address a clearly focused issue? | Yes | Yes | Yes | Yes |
| In terms of the population studied? | Yes | Yes | Yes | Yes |
| In terms of the risk factors studied? | Yes | Yes | Yes | Yes |
| In terms of the outcomes considered? | Yes | Yes | Yes | Yes |
| Is it clear whether the study tried to detect a beneficial or harmful effect? | Yes | Yes | Yes | Yes |
| Was the cohort recruited in an acceptable way? | Yes | Yes | Yes | Can't tell |
| Was the cohort representative of a defined population? | Yes | Yes | Yes | Can't tell |
| Was there something special about the cohort? | No | Yes | No | No |
| Was everybody included who should have been included? | Yes | Yes | Yes | No |
| Was the exposure accurately measured to minimise bias? | Yes | Yes | Yes | Yes |
| Did they use objective measurements? (No if subjective.) | Yes | Yes | Yes | Yes |
| Do the measurements truly reflect what you want them to (have they been validated)? | Yes | Yes | Yes | Yes |
| Were all the subjects classified into exposure groups using the same procedure? | Yes | Yes | Yes | Yes |
| Was the outcome accurately measured to minimise bias? | Yes | Yes | Yes | Yes |
| Did they use objective measurements? (No if subjective.) | Yes | Yes | Yes | Yes |
| Do the measurements truly reflect what you want them to (have they been validated)? | Yes | Yes | Yes | Yes |
| Has a reliable system been established for detecting all the cases (for measuring disease occurrence)? | Yes | Yes | Yes | Yes |
| Were the measurement methods similar in the different groups? | Yes | Yes | Yes | Yes |
| Were the subjects and/or the outcome assessor blinded to exposure (does this matter)? | No | No | No | No |
| Was the follow up of subjects complete enough? | Yes | Yes | Yes | Yes |
| Was the follow up of subjects long enough? | Yes | Yes | Yes | Yes |
| Did the good or bad effects have had long enough to reveal themselves? | Yes | Yes | No | No |
| Do you think the persons that are lost to follow-up had different outcomes than those available for assessment? | No | No | No | No |
| In an open or dynamic cohort, was there anything special about the outcome of the people leaving, or the exposure of the people entering the cohort? | No | No | No | No |
| Do you believe the results? | Yes | Yes | Yes | Can't tell |

|  | **Rakha 2014** | **Sauerbrei 1997** | **Sidoni 2004** | **Suen 2006** |
| --- | --- | --- | --- | --- |
| Did the study address a clearly focused issue? | Yes | Yes | Yes | Yes |
| In terms of the population studied? | Yes | Yes | Yes | Can't tell |
| In terms of the risk factors studied? | Yes | Yes | Yes | Yes |
| In terms of the outcomes considered? | Yes | Yes | Yes | Yes |
| Is it clear whether the study tried to detect a beneficial or harmful effect? | Yes | Yes | Yes | Yes |
| Was the cohort recruited in an acceptable way? | Yes | Yes | No | Can't tell |
| Was the cohort representative of a defined population? | Yes | Yes | Can't tell | Can't tell |
| Was there something special about the cohort? | No | No | No | No |
| Was everybody included who should have been included? | Yes | Yes | No | Can't tell |
| Was the exposure accurately measured to minimise bias? | Yes | Yes | Yes | Yes |
| Did they use objective measurements? (No if subjective.) | Yes | Yes | Yes | Yes |
| Do the measurements truly reflect what you want them to (have they been validated)? | Yes | Yes | Yes | Yes |
| Were all the subjects classified into exposure groups using the same procedure? | Yes | Yes | Yes | Yes |
| Was the outcome accurately measured to minimise bias? | Yes | Yes | Can't tell | Can't tell |
| Did they use objective measurements? (No if subjective.) | Yes | Yes | Yes | Can't tell |
| Do the measurements truly reflect what you want them to (have they been validated)? | Yes | Yes | Can't tell | Can't tell |
| Has a reliable system been established for detecting all the cases (for measuring disease occurrence)? | Yes | Yes | No | No |
| Were the measurement methods similar in the different groups? | Yes | Yes | Yes | Yes |
| Were the subjects and/or the outcome assessor blinded to exposure (does this matter)? | No | No | No | No |
| Was the follow up of subjects complete enough? | Can't tell | Yes | No | Can't tell |
| Was the follow up of subjects long enough? | Can't tell | Yes | No | Yes |
| Did the good or bad effects have had long enough to reveal themselves? | No | No | No | No |
| Do you think the persons that are lost to follow-up had different outcomes than those available for assessment? | No | No | No | No |
| In an open or dynamic cohort, was there anything special about the outcome of the people leaving, or the exposure of the people entering the cohort? | No | No | No | No |
| Do you believe the results? | Yes | Yes | Can't tell | Can't tell |

|  | **Sundquist 1999** | **Wishart 2008** | **Rejali 2015** | **Winzer 2016** |
| --- | --- | --- | --- | --- |
| Did the study address a clearly focused issue? | Yes | Yes | Yes | Yes |
| In terms of the population studied? | Yes | Yes | Yes | Yes |
| In terms of the risk factors studied? | Yes | Yes | Yes | Yes |
| In terms of the outcomes considered? | Yes | Yes | Yes | Yes |
| Is it clear whether the study tried to detect a beneficial or harmful effect? | Yes | Yes | Yes | Can't tell |
| Was the cohort recruited in an acceptable way? | Yes | Yes | Yes | Can't tell |
| Was the cohort representative of a defined population? | Yes | Yes | Yes | Can't tell |
| Was there something special about the cohort? | No | No | Yes | Yes |
| Was everybody included who should have been included? | Yes | Yes | Yes | Can't tell |
| Was the exposure accurately measured to minimise bias? | Yes | Yes | Yes | Yes |
| Did they use objective measurements? (No if subjective.) | Yes | Yes | Can't tell | Yes |
| Do the measurements truly reflect what you want them to (have they been validated)? | Yes | Yes | Can't tell | Yes |
| Were all the subjects classified into exposure groups using the same procedure? | Yes | Yes | Yes | Yes |
| Was the outcome accurately measured to minimise bias? | Can't tell | Yes | Yes | Can't tell |
| Did they use objective measurements? (No if subjective.) | Yes | Yes | Yes | Yes |
| Do the measurements truly reflect what you want them to (have they been validated)? | Can't tell | Yes | Can't tell | Yes |
| Has a reliable system been established for detecting all the cases (for measuring disease occurrence)? | Can't tell | Yes | No | No |
| Were the measurement methods similar in the different groups? | Yes | Yes | Yes | Yes |
| Were the subjects and/or the outcome assessor blinded to exposure (does this matter)? | No | No | No | No |
| Was the follow up of subjects complete enough? | Yes | Yes | Yes | Can't tell |
| Was the follow up of subjects long enough? | Yes | Yes | Yes | Yes |
| Did the good or bad effects have had long enough to reveal themselves? | No | Yes | Yes | Yes |
| Do you think the persons that are lost to follow-up had different outcomes than those available for assessment? | No | No | Can't tell | Can't tell |
| In an open or dynamic cohort, was there anything special about the outcome of the people leaving, or the exposure of the people entering the cohort? | No | No | No | Can't tell |
| Do you believe the results? | Yes | Yes | Yes | Can't tell |

**Appendix 4:** Data extraction procedures

**Extraction of data from survival plots**

Firstly, the graphs were magnified; the survival axis on the graphs were scaled such that one centimetre represented 10% overall survival; the decile of survivors was then read from the graph for each year (from year-one to year –ten); these data on decile of survivors per year recorded in a two-by-two matrix; the distance from the origin of the graph was measured for every year for every NPI group and converted to the percentage surviving each year (assuming one centimetre = 10% overall survival).

**Appendix 5:** Reported five and ten-year survival per study dataset

| **5 and 10-year survival in each dataset** | | |  |
| --- | --- | --- | --- |
| **Dataset Source** | **5-year survival (%)** | **10-year survival (%)** | **Number in group** |
| **Galea 1992 (UK)** |  |  |  |
| NPI 1 | 92.54 | 83.16 | 470 |
| NPI 2 | 70.94 | 51.87 | 879 |
| NPI 3 | 24.56 | 13.52 | 280 |
| **Balslev 1994 (Denmark)** | | | |
| NPI 1 | 91.53 | 79.05 | 2494 |
| NPI 2 | 75.02 | 56.01 | 5245 |
| NPI 3 | 37.94 | 24.47 | 1410 |
| **Hansen 2000 (Denmark)** | | | |
| NPI 1 | 95.97 | 85.89 | 163 |
| NPI 2 | 77.51 | 63.1 | 250 |
| NPI 3 | 34.13 | 39.02 | 122 |
| **D'Eredita 2001 (Italy)** | | | |
| NPI 1 | 96.4 | 86.49 | 110 |
| NPI 2 | 76.38 | 69.85 | 198 |
| NPI 3 | 48.96 | 39.58 | 94 |
| **Sidoni 2004 (Italy)** |  |  |  |
| NPI 1 | 92.59 | 85.19 | 27 |
| NPI 2 | 75 | 60 | 39 |
| NPI 3 | 62.5 | 50 | 16 |
| **Callagy 2006 (Canada)** | | | |
| NPI 1 | 88.24 | 73.53 | 34 |
| NPI 2 | 77.12 | 58.05 | 236 |
| NPI 3 | 56.45 | 37.98 | 287 |
| **Suen 2006 (Hong Kong)** | | | |
| NPI 1 | 81.25 | N/A | 32 |
| NPI 2 | 81.63 | N/A | 97 |
| NPI 3 | 53.85 | N/A | 52 |
| **Jarman 2008 (UK)** |  |  |  |
| NPI 1 | 95.77 | N/A | 401 |
| NPI 2 | 83.21 | N/A | 131 |
| NPI 3 | 40.74 | N/A | 27 |
| **Wishart 2008 (UK)** |  |  |  |
| NPI 1 | 94.05 | N/A | 1765 |
| NPI 2 | 85.11 | N/A | 1820 |
| NPI 3 | 58.01 | N/A | 512 |
| **Blamey 2010 (10 EU countries)** | | | |
| NPI 1 | 96.17 | 90.12 | 4128 |
| NPI 2 | 88.31 | 77.4 | 4235 |
| NPI 3 | 67.74 | 50.42 | 1183 |
| **Allgood 2011 (UK)** |  |  |  |
| NPI 1 | N/A | 88.86 | 4855 |
| NPI 2 | N/A | 69.93 | 4746 |
| NPI 3 | N/A | 37.11 | 2487 |
| **Bundred 2013 (UK)** |  |  |  |
| NPI 1 | 98.97 | 95.62 | 388 |
| NPI 2 | 91.61 | 83.45 | 415 |
| NPI 3 | 76.74 | 40.7 | 85 |
| **Fong 2015 (UK)** |  |  |  |
| NPI 1 | 93.04 | 85.5 | 859 |
| NPI 2 | 86.75 | 76.31 | 536 |
| NPI 3 | 83.55 | 69.74 | 151 |
| **Rejali 2015 (Iran)** |  |  |  |
| NPI 1 | 96.67 | 96.67 | 30 |
| NPI 2 | 82.05 | 73.5 | 116 |
| NPI 3 | 73.26 | 59.3 | 87 |

Note: Studies listed in order of publication by date

N/A: means ‘not applicable’ when studies did not provide data for both 5 and 10-year survival.

NPI 1 – Good Prognostic Group

NPI 2 – Moderate Prognostic Group

NPI 3 – Poor Prognostic Group

1. Breast cancer was assumed a cause of death only for the women who died with known metastases of their disease. Survival estimates from this study are therefore less comparable with the other studies. [↑](#footnote-ref-1)
2. Follow-up completed only by 89% of patients, but there is no reason to assume that attrition was selective in relation to outcomes. [↑](#footnote-ref-2)
3. Follow-up period was only 2 years for the patients from NPI category 1. This is not likely to affect the results of meta-analysis, because the prognosis for this group is very good. [↑](#footnote-ref-3)
